# Factors affecting bowel preparation adequacy and procedural time

**DOI:** 10.1002/jgh3.12241

**Published:** 2019-08-20

**Authors:** Mohammadali Zad, Cuong N Do, Aaron Heffernan, Lucy Johnston, Mohammed Al‐Ansari

**Affiliations:** ^1^ Gastroenterology, Logan Hospital Brisbane Queensland Australia; ^2^ General Medicine, Princess Alexandra Hospital Brisbane Queensland Australia; ^3^ School of Medicine Griffith University Gold Coast Queensland Australia; ^4^ Paediatrics, Monash Hospital Melbourne Victoria Australia; ^5^ Gastroenterology, Ballarat Hospital Ballarat Victoria Australia

**Keywords:** bowel preparation, colonoscopy, procedure time, runway time

## Abstract

**Background and Aim:**

Poor bowel preparation results in difficult colonoscopies, missed lesions, and repeat procedures. Identifying patient risk factors for poor bowel preparation, such as prolonged runway time and prolonged cecal intubation, will aid in interventions prior to a procedure.

**Methods:**

This was a retrospective, single‐center analysis of 3 295 colonoscopies performed between May 2012 and November 2014. Indications for colonoscopy included gastrointestinal bleed and anemia, change in bowel habits, for screening, and others (including planning re‐anastomoses, abdominal distension, family history and angioectasias). Data were collected from medical charts and endoscopy reports. Comparisons between patient factors and runway time were made with adequacy of bowel preparation as the primary outcomes.

**Results:**

Male and diabetic patients had statistically higher rates of inadequate bowel preparation and prolonged cecal intubation times. A previous history of abdominal surgery also demonstrated prolonged cecal intubation. A runway time of ≤7.63 h was associated with higher rates of adequate bowel preparation by multivariate analysis. The optimal time frame is 3–6 h for the highest success rates.

**Conclusion:**

Patient risk factors for inadequate bowel preparation or prolonged cecal intubation should signal clinicians to intervene prior to colonoscopy. A runway time between 3 and 6 h is optimal for adequate bowel preparation. This may involve further patient education, along with work flow optimization, to facilitate ideal runway times. Future studies should explore how to avoid repeat endoscopies using protocols enforcing this timeframe.

## Graphical abstract

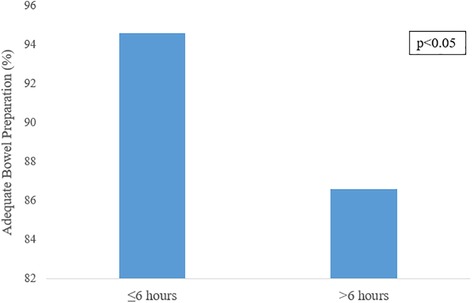

Optimizing runway time plays a critical role in adequate bowel preparation for colonoscopy. Identifying methods of good bowel preparation in patients is ideal to prevent repeat procedures.

## Introduction

Colonoscopy is the current gold standard for the investigation and removal of precursor adenomatous polyps. The effectiveness of colonoscopy depends on the adequacy of bowel preparation, which itself is influenced by the preparation agent and timing of dose. Patient factors also affect adequacy of bowel preparation, including gender, body mass index (BMI), diabetes history, smoking history, and history of surgery.^1^, ^2^

Adequacy of bowel preparation is critical to the effectiveness of a colonoscopy; however, bowel preparation is inadequate in more than 25% of all colnoscopies.^3^ A study (*n* = 373) examining repeat colonoscopies due to inadequate bowel preparation found that at least one adenoma was detected in 33.8%, and a high risk state was detected in 18%.^4^ Inadequate bowel preparation is also associated with increased procedure time, adverse events, and aborted procedures.^5^, ^6^ This invariably incurs costs to the health‐care system, with economic models in the United States concluding that screening colonoscopies are not cost‐effective as long as the inadequate bowel preparation rate is greater than 13%.^7^ Hence, it is imperative to improve the quality of bowel preparation.

Runway time is defined as the interval between the last dose of preparation agent and the start of the colonoscopy. Optimizing runway time prior to colonoscopy is likely an underappreciated means to improve the rates of adequate colonoscopy preparation. A meta‐analysis also found that, as runway time increased, the gain in bowel preparation quality of split dosing over nonsplit dosing decreased. It found that the superior quality of bowel preparation from split dosing was maintained when the colonoscopy was performed within 3 h of the last dose of preparation agent but decreased after 4–5 h. The authors suggested that it was not the type or dose of laxative but the golden 5 h that was critical to the colonoscopy of a clean colon.^8^ This idea warrants further exploration as it could significantly affect the quality of bowel preparation in Australian colonoscopies. The American College of Gastroenterology (ACG) recommends a period of 4–6 h before the start of the colonoscopy from the completion of the last dose of bowel prep.^9^ Studies on runway time are largely based in the United States, and there is a need to adapt these findings to Australian practice.

Patient factors also play a significant role in bowel adequacy. Older age (>50 years), male gender, and higher BMI have occasionally been associated with inadequate bowel preparation in the literature. Patients with comorbidities, such as diabetes and smoking, have also been associated with poorer bowel adequacy prior to colonoscopy.^9^, ^10^, ^11^, ^12^ A small study (*n* = 99) of diabetic patients showed that only 64% had good or better bowel preparation compared to 97% of nondiabetic patients.^13^ Previous surgical history has been associated with unsatisfactory bowel preparation. A prospective cohort study (*n* = 362) showed that 64% of patients with previous gastric resection and 59.7% of patients with previous colonic resection had unsatisfactory bowel preparation.^14^ The importance of identifying patient risk factors lies in the potential benefit of tailored protocols that aim to improve preparation efficacy and tolerability. There remain a few studies that attempt to define the clinical predictors of inadequate bowel preparation.

Furthermore, split‐dose preparations, whereby a portion of preparation is given on the day of the examination, have in recent years become the standard for clinical practice. Split‐dose preparations increase the likelihood of adequate bowel preparation, as well as improve patient compliance and patient tolerance.^15^, ^16^ However, it is not yet well understood how patient risk factors and runway time of the preparation dose interact with split‐dosing regimens.

Patient factors have also been reported to affect procedural time. Cecal intubation time is often used as a surrogate to estimate the difficulty of a colonoscopy. Shorter cecal intubation times have been associated with increased detection rates of polyps. Many patient factors, such as age, obesity, or poor bowel preparation, have been shown make it difficult for endoscopists to achieve cecal intubation, thus prolonging procedural time.^17^, ^18^ A prospective study (*n* = 1 043) showed that the mean number of adenomas detected per patient decreased across increasing cecal intubation time by 7–11% per time quartile.^19^ Thus, it is important to consider which of these factors may be clinically significant considerations prior to colonoscopy.

## Methods

### *Study design*

The study was a retrospective, single‐center analysis of 3 295 colonoscopies performed between May 2012 and November 2014 in a regional hospital in Australia. Seventeen experienced endoscopists performed and reported on the colonoscopies to minimize interobserver variation. Patients were provided with written and verbal instructions from endoscopy nurses at the time of booking regarding the bowel preparation regimen and fasting times based on the time of their procedure. Only patients who reported compliance with bowel preparation were included in the study. The types of bowel preparation agents used in the study were Picoprep, Picolax, and Prekit‐C.

### *Study population*

**Figure 1:**
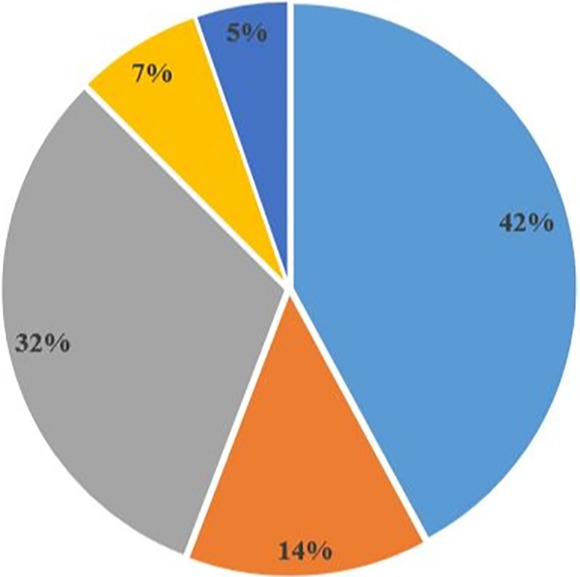
Indications for colonoscopy. (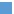), lower gastrointestinal bleed and anemia; (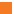), change in bowel habits; (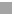), surveillance and screening; (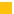), abdominal pain; (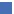), others.

Study patients included men and women ≥18 years of age. Indications for colonoscopy included lower gastrointestinal bleed and anemia (42.1%), change in bowel habits (13.7%), surveillance and screening (31.7%), abdominal pain (7.2%), and other abdominal symptoms (5.3%). This is shown in Figure 1.

Patients were also split into an AM (07:30–12:00 h) or PM (after 12:00 h) group depending on whether they had the procedure in the morning or afternoon, respectively. A split‐dosing regime was implemented in both groups.

### *AM group (07:30–12:00 h)*

Two sachets of bowel preparation agent were ingested the day prior to the procedure starting around 15.00, with at least 2 h between each sachet. The third sachet was ingested at 05:00 on the day of procedure. Solids were ceased after 08.00 the day prior to the procedure, and clear fluids were consumed until 24.00 h (midnight).

### *PM group (after 12:00 h)*

Two sachets of bowel preparation were ingested the day prior, starting at 17:00 h, with at least 2 h between each sachet. The third sachet was ingested at 10:00 h on the day of the procedure. Solids were ceased after 08:00 h the day prior, and clear fluids were continued until 4 h prior to the procedure.

### *Data collection*

Patient variables collected included: patient demographics (age and gender), colonoscopy indication, timing of bowel preparation, fasting regimen, medical history (history of coronary artery disease, diabetes, smoking history), and surgical history (history of bowel, colon, and pelvic surgery). In this study, a diagnosis of chronic obstructive pulmonary disease (COPD) is used as a surrogate for smoking history. Procedure variables collected included: assessment of bowel adequacy (by Ottawa Preparation Scale) and procedural time (cecal intubation, withdrawal).

The runway time was calculated as the difference between the last dose of preparation agent and the start of the colonoscopy in terms of hours.

The primary outcome assessed was the quality of bowel preparation as reported by endoscopists in accordance with the Ottawa Bowel Preparation Scale,^20^ a validated bowel preparation scale. The instrument requires endoscopists to rate the adequacy of three segments (right colon, mid colon, and recto sigmoid colon) on a scale: 0 = excellent (mucosa clearly visible, almost no stool/fluid residual), 1 = good (some stool/fluid residue but mucosa visible even without suctioning), 2 = fair (some turbid stool/fluid but mucosa visible with some suctioning), 3 = poor (stool obscuring view of mucosa but reasonable view obtained with suctioning and washing), and 4 = inadequate (solid stool obscuring mucosa and not cleared with suctioning and washing).

To simplify the presentation of data, Ottawa Preparation scale scores of 0 (excellent), 1 (good), and 2 (fair) were assigned to Group 1 (adequate bowel preparation) and scores of 3 (poor) and 4 (inadequate) were assigned to Group 2 (inadequate bowel preparation).

Secondary outcomes of procedural times were also collected. Cecal intubation time (minutes) was defined as the reported time taken to pass the colonoscope proximal to the ileocecal valve (the most reliable cecal landmark). Withdrawal, or cecal extubation time, was defined as the reported time (minutes) taken from cecal intubation to withdrawal of the colonoscope from the anus. Cecal intubation and cecal extubation time did not include intervention times (e.g. polypectomy).

### *Data analysis*

The patient factors analyzed include patient demographics, age (age 50 and under or over 50 years old), gender (male *vs* female), colonoscopy indication and timing of bowel preparation and fasting regimen, medical history (history of coronary artery disease, diabetes, smoking history), and surgical history (history of bowel, colon, and pelvic surgery). How these patient factors affected the frequency at which patients achieved adequate bowel preparation (Ottawa Preparation Scale 0, 1, or 2) was assessed for significance using the Pearson Chi‐square test.

Similarly, the ideal time frame from the last dose of preparation agent to the start of colonoscopy (runway time) when divided into categorical blocks of <3‐h, >8 ‐h, and 1‐h time frames in between that achieved the most outcomes of adequate bowel preparation was analyzed using the Pearson Chi‐Square test. A *P* value <0.05 was considered statistically significant. The differences between patients in the AM group and PM group regarding their endoscopy procedure were also compared for significance.

Multivariate analysis was performed using classification and regression decision tree models and binomial logistic regression using R. Both model parameters were optimized using fivefold cross validation. If an optimal dividing point for a continuous variable (such as runway time) was identified using decision tree modeling, this was incorporated into the logistic regression model. The final model was selected considering the Akaike information criterion (AIC) and the area under the receiver operator curve. Summary results include the odds ratio (OR) and 95% confidence interval (CI).

**Figure 2:**
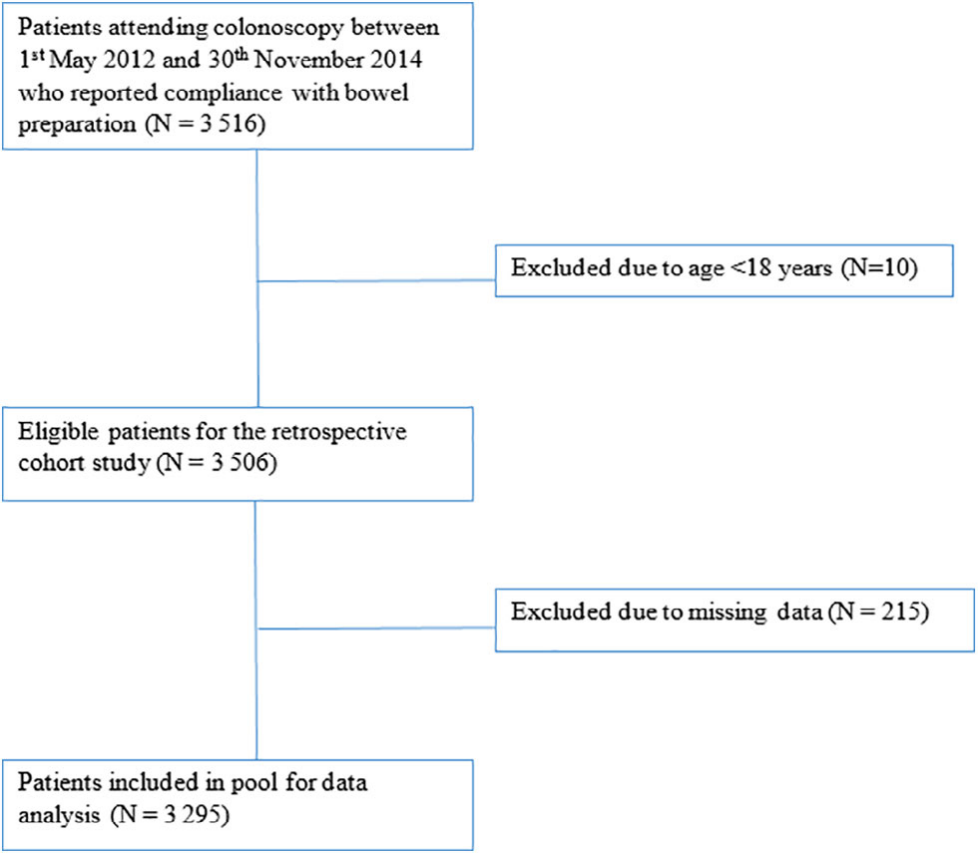
Exclusion flow chart.

The secondary outcomes investigated were how procedural time (cecal intubation and cecal extubation times) was influenced by the same patient factors. This was achieved through the Mann–Whitney Test with a *P*‐value <0.05 to define statistical significance. The results were represented as median and interquartile range (IQR). When missing data were encountered, the patient was excluded from data analysis for that comparison. Shown in Figure 2 is the Exclusion Flow Chart.

## Results

A total of 3 295 patients ranging from 19 to 94 years of age were recruited for this study. These patients were recruited consecutively from a list of those undergoing colonoscopy from May 2012 to November 2014 in a regional Australian hospital.

### *Detection rate of polyps*

**Figure 3:**
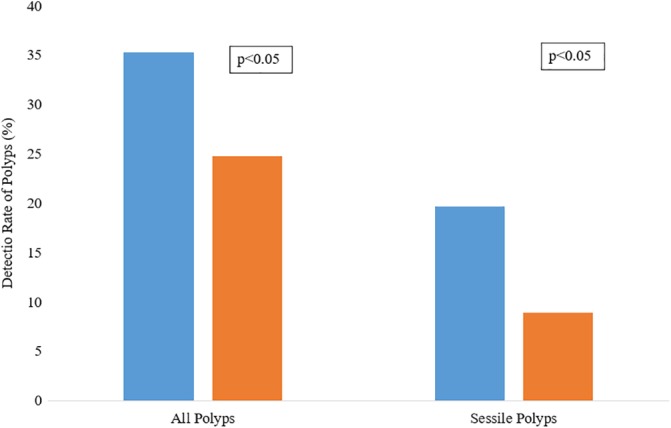
Detection rate of all polyps and sessile polyps. (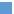), Adequate bowel preparation; (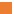), inadequate bowel preparation.

The detection rate of polyps in this study was 34.36%. In patients with adequate bowel preparation (*n* = 2 992), the detection rate of polyps was found to be 35.3% (*n* = 1 057). In patients with inadequate bowel preparation (*n* = 303), the detection rate was 24.75% (*n* = 303). This represented a statistically significant higher rate of polyp detection in patients with adequate bowel preparation (*P* < 0.000221). The detection rate of sessile polyps in patients with adequate bowel preparation was 19.69% (*n* = 589). Comparatively, in patients with inadequate bowel preparation, it was 8.91% (*n* = 27), which represented a statistical difference (*P* < 0.0362). This is illustrated in Figure 3 (Table 1).

**Table 1:** Patient demographics (*n* = 3 295)

Gender	
Male	1 798 (55%)
Female	1 497 (45%)
Age	
≤50	932 (28%)
>50	2 356 (72%)
Body mass index	
Underweight, <18.5	72 (2.4%)
Healthy weight, 18.5–24.9	780 (26.2%)
Overweight, 25–29.9	994 (33.4%)
Obese, >30	1 128 (38%)
Diabetes mellitus history†	
Yes	417 (13%)
No	2 875 (87%)
Ischemic heart disease history	
Yes	292 (9%)
No	3 003 (91%)
Chronic obstructive pulmonary disease history	
Yes	158 (5%)
No	3 137 (95%)
Abdominal surgery history‡	
Colon	133 (12%)
Bowel	75 (7%0
Pelvic	20 (2%)
Hysterectomy	229 (20%)
Other§	419 (38%)
No surgery	239 (21%)

† Diabetes Mellitus Type 1 and Type 2.

‡ Only included where the patient was specifically asked about abdominal surgery history and recorded.

§ Other abdominal surgery, including hernia repair and laparoscopic procedure.

### *Patient factors*

Results showed that only 89.6% of patients >50 years (*n* = 2 356) had adequate bowel preparation compared to 93.3% of patients ≤50 years (*n* = 932) (*P* < 0.05). The OR of patients ≤50 years old having adequate bowel preparation compared to >50 years was 1.62 (95% CI 1.21–2.16). In this study, only 87.1% of diabetic patients (*n* = 417) achieved adequate bowel preparation compared to 91.3% of nondiabetic patients (*n* = 2 875) (*P* = 0.005). The OR of nondiabetics having adequate bowel preparation compared to diabetics was 1.57 (95% CI 1.15–2.15). Table 2 shows the comparisons between all patient factors in terms of adequacy of bowel preparation achieved.

**Table 2:** Patient factors affecting likelihood of adequate bowel preparation

Bowel preparation	Adequate (%)	Inadequate (%)	*P* value
Gender			
Male, *n* = 1 798	91.2	8.8	0.374
Female, *n* = 1 497	90.3	9.7	
Age			
≤50, *n* = 932	89.6	10.4	<0.05
>50, *n* = 2 356	93.3	6.7	
BMI†			
Underweight, *n* = 72	86.1	13.9	0.425
Normal weight, *n* = 780	91	9	
Overweight, *n* = 994	91.2	8.8	
Obese, *n* = 1 128	91.8	8.2	
DM			
Yes, *n* = 417	87.1	12.9	0.005
No, *n* = 2 875	91.3	8.7	
IHD			
Yes, *n* = 292	89.7	10.3	0.504
No, *n* = 3 003	90.9	9.1	
COPD			
Yes, *n* = 158	88	12	0.207
No, *n* = 3 137	90.9	9.1	
Abdominal Surgery History‡			
Colon, *n* = 133	89.5	10.5	0.785
Bowel, *n* = 75	93.3	6.7	
Pelvic, *n* = 20	95	5	
Hysterectomy, *n* = 229	91.7	8.2	
Other§, *n* = 419	92.1	7.9	
No surgery, *n* = 239	91.6	8.4	

† BMI <18.5 (underweight), BMI 18.5–24.9 (normal weight), BMI 25–29.9 (overweight), BMI >30 (obese).

‡ Only included where the patient was specifically asked about abdominal surgery history and recorded.

§ Other abdominal surgery, including hernia repair and laparoscopic procedure.

BMI, body mass index; COPD, chronic obstructive pulmonary disease; DM, diabetes mellitus (1 and 2); IHD, ischemic heart disease.

Multivariate analysis showed that increasing age was weakly associated with inadequate bowel preparation (OR 1.01, 95% CI 1.01–1.02), as was a previous diagnosis of diabetes mellitus (OR 1.54, 95% CI 1.14–2.09). Time of last oral intake of <6.88 h was associated with improved rates of adequate bowel preparation using decision tree analysis and logistic regression (OR 1.05, 95% CI 1.02–1.09). Gender, a previous diagnosis of COPD, previous surgical history, indication for colonoscopy, and BMI were not significantly associated with bowel preparation adequacy.

### *Procedure factor*

**Figure 4:**
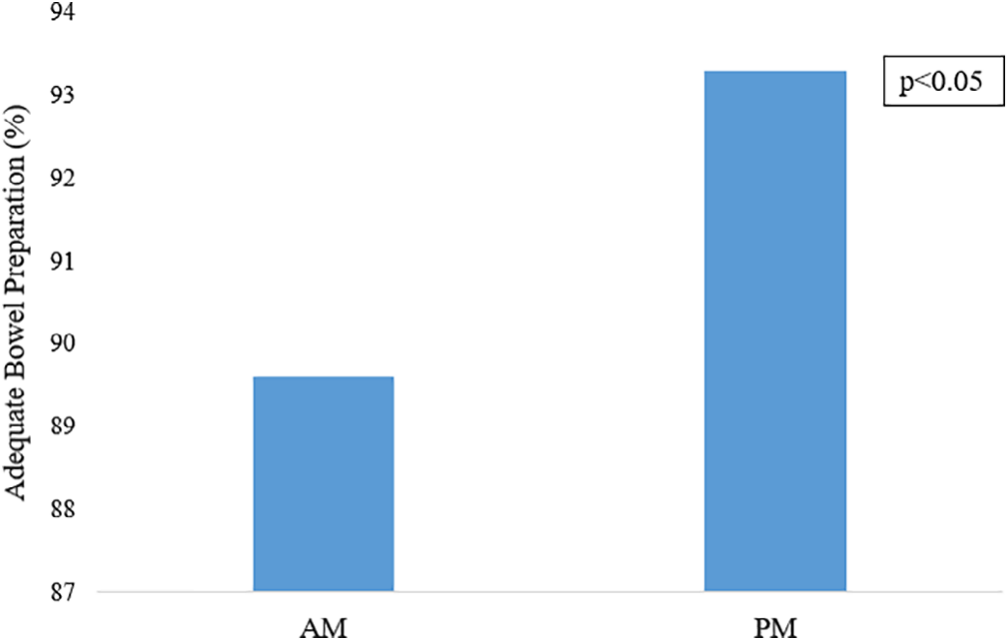
Procedure timing affecting likelihood of adequate bowel preparation.

Of the patients who were included in the AM group (*n* = 2 122), only 89.3% had adequate bowel preparation. In comparison, 93.3% of patients undergoing a colonoscopy in the PM group (*n* = 1 103) had adequate bowel preparation (*P* < 0.05). This is illustrated in Figure 4. The OR of PM group patients having adequate bowel preparation compared to AM group patients was 1.85 (95% CI 1.39–2.46). Through multivariate analysis, a PM procedure time was associated with reduced rates of inadequate bowel preparation (OR 0.66, 95% CI 0.54–0.82).

### *Runway time*

**Figure 5:**
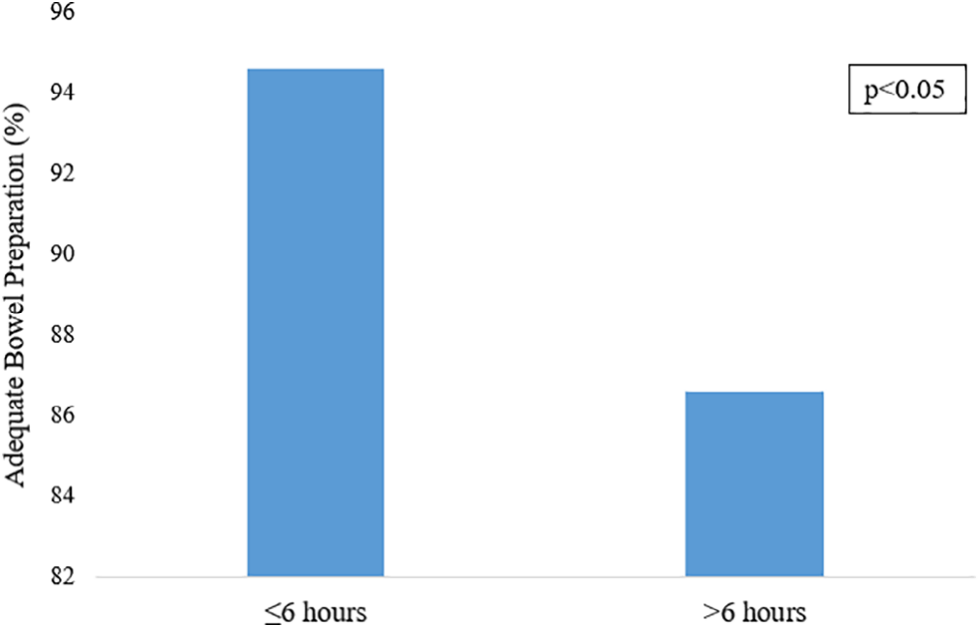
Runway time affecting adequate bowel preparation.

Runway time was defined as the interval between the last dose of preparation agent and the start of colonoscopy. This study found that 94.6% of patients with a runway time of 6 h or less (*n* = 1 741) achieved adequate bowel preparation using univariate analysis. Comparatively, only 86.6% of patients with a runway time greater than 6 h (*n* = 1 454) achieved adequate bowel preparation (*P* < 0.05). This is shown in Figure 5. This represented an OR of achieving adequate bowel preparation with a runway time of 6 h or less *versus* more than 6 h of 2.7 (95% CI 2.1–3.5).

**Figure 6:**
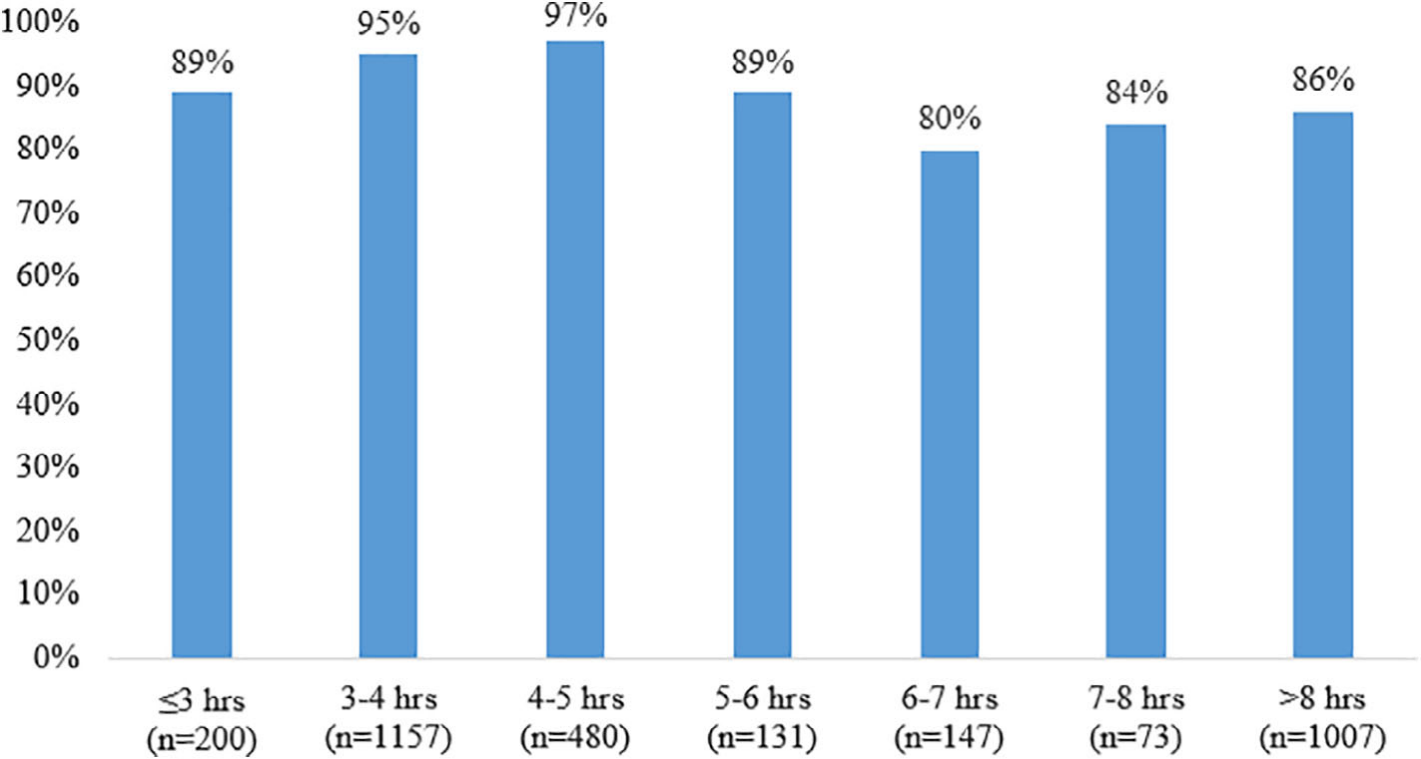
Adequate bowel preparation in hourly blocks.

Figure 6 shows the rate of patients achieving adequate bowel preparation by hourly blocks of runway time. Patients with a runway time of 4–5 h (*n* = 480) achieved the highest rate of bowel adequacy (97%) of all the time blocks.

Multivariate analysis using a decision tree showed that a runway time of >7.63 h was associated with increased inadequate bowel preparation. Adjusting the runway time to >7.63 and < 7.63 h in the logistic regression analysis also showed increased inadequate bowel preparation (OR 3.10, 95% CI 2.38–4.04). A further logistic regression model incorporating runway time as a categorical variable separated into >6 and <6 h was shown to be an inferior model when compared with the previous separation of runway time into >7.63 h and <7.63 h (AIC 2 498 *vs* 2 521). Univariate and multivariate analysis of variables of interest in this study are shown in Table 3.

**Table 3:** Univariate and multivariate logistic regression of factors for inadequate bowel preparation

	Univariate analysis		Multivariate analysis	
Variable	OR (95% CI)	*P* value	OR (95% CI)	*P* value
Age	1.008 (1–1.02)	0.047	1.01 (1.00–1.02)	<0.001
Gender (if female)	0.89 (0.71–1.13)	0.35	0.87 (0.71–1.07)	0.18
BMI	0.99 (0.97–1.01)	0.23	1.01 (0.99–1.02)	0.48
DM (if yes)	1.54 (1.2–2.12)	0.008	1.54 (1.14–2.08)	<0.001
IHD (if yes)	1.18 (0.8–1.75)	0.4	1.30 (0.91–1.87)	0.15
COPD (if yes)	1.46 (0.9–2.37)	0.13	1.08 (0.66–1.75)	0.77
Runway Time	2.7 (2.09–3.47)	<0.001	3.37 (2.56–4.44)	<0.001
AM_PM	0.51 (0.42–0.62)	<0.001	0.70 (0.57–0.87)	<0.001
Last oral intake	1.16 (1.13–1.19)	<0.001	1.05 (1.01–1.09)	0.0127

† Runway time – univariate analysis (if >6 h), multivariate analysis (if >7.63 h).

BMI, body mass index; CI, confidence interval; COPD, chronic obstructive pulmonary disease; DM, diabetes mellitus type 1 and 2; IHD, ischemic heart disease; OR, odds ratio.

### *Procedural time*

This study examined the procedural time, represented by cecal intubation and extubation time and described in Table 4, as secondary outcomes. Median cecal intubation time in minutes for men was 7 (IQR 5–11), which was longer than that for women, which was 6 (IQR 5–10) (*P* = 0.001). However, men had a shorter median cecal extubation time at 6 min (IQR 4–10) compared to women at 7 min (IQR 5–11) (*P* = 0.001). Patients with diabetes had a median cecal intubation time of 8 min (IQR 5–14), which was longer compared to nondiabetics at 6 min (IQR 5–10) (*P* = 0.001). Patients with ischemic heart disease (IHD) also had a longer median cecal intubation time of 7 min (IQR 5–12) compared to those without IHD at 6 min (IQR 5–10) (*P* = 0.034). Finally, patients with a history of abdominal and pelvic surgery had a statistically significant longer cecal intubation time compared to those without surgery (*P* = 0.03). When runway time was 6 h or less, patients had a statistically shorter extubation time of 6 min (IQR 4–11) compared with 7 min (IQR 4–12) (*P* < 0.0001) in those with runway time greater than 6 h.

**Table 4:** Clinical factors affecting intubation and extubation time

	Intubation time (min), median (IQR)	*P* value	Extubation time, (min), median (IQR)	*P* value
Gender				
Male	7 (5–11)	0.001	6 (4–10)	0.001
Female	6 (5–10)		7 (5–11)	
BMI†				
Underweight	7.3 (4–13)	0.18	6 (4–10)	0.1
Normal weight	6 (5–10)		6 (4–10)	
Overweight	6 (5–10.3)		7 (4–11)	
Obese	7 (5–11)		7 (5–11)	
DM		0.001		0.11
Yes	8(5–13)		7 (5–11)	
No	6 (5–10)		7 (4–11)	
IHD		0.034		0.003
Yes	7 (5–12)		8 (5–12)	
No	6 (5–10)		7 (4–10)	
COPD		0.2		0.1
Yes	7 (5–11)		7 (5–10)	
No	6 (5–11)		7 (4–11)	
Surgical history‡		0.03		0.06
Colon	6 (5–9)		7 (4–10)	
Bowel	6 (4–9)		6 (4–10)	
Pelvic	6 (4–7)		5(4–6)	
Hysterectomy	7 (5–10)		6 (4–10)	
Other§	7 (5–12)		7 (4–10)	
No surgery	6 (5–10)		7 (5–11)	
Runway time				
6 h or less	8 (5–12)	0.18	6 (4–11)	<0.0001
More than 6 h	7 (5–12)		7 (4–12)	

† BMI <18.5 (underweight), BMI 18.5–24.9 (normal weight), BMI 25–29.9 (overweight), BMI >30 (obese).

‡ Only included where the patient was specifically asked about abdominal surgery history and recorded.

§ Other abdominal surgery, including hernia repair and laparoscopic procedure.

BMI, body mass index; COPD, chronic obstructive pulmonary disease; DM, diabetes mellitus (1 and 2); IHD, ischemic heart disease; IQR, interquartile range.

## Discussion

Adequacy of bowel preparation is critical for successful colonoscopy procedures. It allows for clear visualization of potential lesions. While patient compliance with bowel preparation is the most significant factor in determining adequacy, other considerations, such as optimization of runway time and patient demographics, also play an important role.

The primary outcome in this study was adequacy of bowel preparation, which represents a higher likelihood of detecting polyps on colonoscopy. The detection rate of polyps was statistically higher in patients with adequate bowel preparation. This echoes existing literature and emphasizes the importance of bowel preparation in preventing missed polyps. Our study suggested that up to 10.55% of polyps could be missed with inadequate bowel preparation. Furthermore, 10.78% of sessile polyps, which are notably difficult to detect on colonoscopy as they lie flat against colonic mucosa, could be missed with inadequate bowel preparation. However, our study did not delineate on the types of polyps likely to be missed. A meta‐analysis by Sulz *et al*. concluded that suboptimal preparation could reduce the detection of early polyps, which are more difficult to visualize, by more than 20%. Detection of advanced, larger lesions was less affected in their analysis but was still greater in patients with optimal bowel preparation.^21^, ^22^ Future studies could examine the histological significance of polyps not detected due to inadequate bowel preparation.

A runway time of ≤6 h was significantly (*P* < 0.05) associated with adequate bowel preparation. When a multivariate analysis was applied, taking into consideration patient factors, this optimal runway time was ≤7.63 (*P* < 0.001). A multivariate analysis found older patient age, diabetes status, runway time >7.63 h, and AM procedures to be statistically associated with inadequate runway time (*P* < 0.05). This is shown in Table 3. With the highest impacting OR, runway time (OR 3.37, 95% CI 2.46–4.44) played the most important role in determining inadequate bowel preparation. Runway time is a modifiable factor by clinicians. Although logistical constraints exist in the health‐care system, a standardized and protected time frame between the last dose and procedure time would reduce missed polyps from inadequate bowel preparation. The presence of diabetes was the second most impactful determining factor (OR 1.54, 95% CI 1.14–2.08). This represents a vulnerable group of patients who may benefit from further clinician intervention to improve bowel preparation and polyp detection.

This study found that patients 50 years or younger and nondiabetic patients had a statistically higher frequency of adequate bowel preparation (rated 0, 1, or 2 on Ottawa Preparation Scale overall) as rated by endoscopists. This reinforces existing literature, which lists both older age and diabetic status as risk factors for poor bowel preparation adequacy.^10^, ^11^, ^12^ Aging causes degeneration of the autonomic nervous system that controls enteric smooth muscles. Furthermore, older patients tend to be more immobile. Diabetes is thought to cause neuropathic injury, including demyelination and axonal degeneration, to those same nerves. Both can result in constipation, which leads to poor bowel preparation in these patients.^23^, ^24^, ^25^, ^26^ The presence of risks factors for inadequate bowel preparation helps inform the decision of the regime and may indicate to the clinician that stricter bowel preparation is needed. These patients should also be targeted for further support and education, emphasizing the importance of compliance to avoid repeat colonoscopy. A study by Prakash *et al*. suggested that instructional bowel preparation videos could significantly improve patient adherence and quality.^27^

Nonsignificant differences were seen in male *versus* female patients, different BMI, history of IHD, history of COPD, and history of abdominal surgery in terms of adequate bowel preparation. Previous literature identified male gender and obesity as risk factors for poor bowel preparation.^28^, ^29^ Male (55%) and female (45%) patients in this study were evenly proportioned, and no statistically significant difference was detected between the quality of bowel preparation. This may reflect differences in the diet and level of physical activity between the Australian population sampled in this study and the American populations of prior studies.

Patients in the PM group had statistically higher rates of adequate bowel preparation related to runway time optimization. Runway time was found to ideally be ≤6 h by univariate analysis and ≤7.63 by multivariate analysis. The ACG recommends a runway time of 4–6 h; however, these are based on American studies, and it was important to reaffirm these results in an Australian‐based study. It is important to note that bowel preparation did not linearly improve with lower runway time. As seen in Figure 6, the highest rates of adequate bowel preparation were between 3 and 6 h.

A series of meta‐analyses by Bucci *et al*. showed that optimizing runway time had a more critical role in improving the quality of split‐dosing regimens than the type or dose of bowel preparation agent.^8^ Adequate bowel preparation minimizes the risk of missing polyps, particularly small or sessile lesions, and prevents the need for a repeat endoscopy, saving time and money. Despite this, an optimal time frame of 3–6 h can be challenging given the heavy workloads of endoscopy suites in Australian hospitals. Global cancer statistics show that Australia, alongside New Zealand, has the highest incidence of colorectal cancer in the world.^30^ Future studies shoulder explore repeat colonoscopies that are avoided as a result of protocols mandating optimal runway time for patients. Ideally, benefits would be seen in a reduced burden on the health economy and reduced patient wait times for procedures.

Cecal intubation is a surrogate for the difficulty of colonoscopy. This secondary outcome was explored because prolonged cecal intubation times have been associated with decreased polyp detection and increased risk of adverse events from the procedure or sedation.^17^, ^18^, ^31^ Understanding the factors for challenging colonoscopies is important to stratify the risks of missed lesions in patients. This study showed that cecal intubation was statistically longer for male, diabetic patients and patients with a history of abdominal surgery. This reinforces existing literature and is believed to relate to the rate of adequate bowel preparation. Poor bowel preparation has been associated with longer cecal intubation.^20^, ^32^, ^33^, ^34^ Furthermore, the presence of adhesions from abdominal surgery can lead to constipation and difficulty maneuvering during colonoscopy. A runway time of 6 h or less did not improve cecal intubation time in this study; however, cecal extubation was notably shorter, which suggests an overall faster procedure. Although nonsignificant, patients with a BMI classified as underweight (<18.5) and obese (>30) had longer cecal intubation times. This is because leaner patients have smaller abdominal cavities in which to maneuver, whilst patients with high BMI are difficult to reposition or apply abdominal pressure.^35^

This study was a retrospective clinical audit of patient data, designed to assess patterns during the process of patient care. As such, it is dependent on the accuracy and quality of information recorded in patient records. The reliance on an accurate history by individual clinicians collecting them was diluted to some extent by the large sample size. Although some patient characteristics are balanced in the sample, for example, males and females (55% *vs* 45%), others leaned heavily (72% of patients were >50 years old, whereas only 28% were age ≤50). Although the comparisons in those cases have unbalanced numbers, this can be thought to better represent the actual population undergoing colonoscopies. Thus, the results of this study are generalizable to the larger Australian population.

A major advantage of this study is that it encompasses a large number of patients undergoing colonoscopies (*n* = 3 295) during the 2‐year period. The only exclusion criterion was age <18 years and noncompliance with bowel preparation, which allowed for a high volume of patients to be audited. This avoids the selection bias inherent with smaller sample sizes. However, a major weakness of retrospective data collection is its reliance on existing information recorded in databases. Our study had 215 patients who were excluded due to missing data. Misclassification bias is a significant risk in retrospective cohort studies, which rely on data being correctly recorded for the accuracy of its conclusions.

Retrospective studies are also vulnerable to confounding variables. Double blinding and randomization are not possible to use in retrospective cohort studies. As such, at best, only an association between variables can be established and not causation. In future study designs, accounting for these variables with their own subanalysis would help mitigate and understand their effect. A prospective study whereby patients are randomly assigned to different runway times would add further weight to the argument.

Another potential source of bias was in the subjectivity of individual endoscopists. There were 17 experienced endoscopists who reported on the quality of bowel preparation through the validated Ottawa Score. This helped minimize bias from individual endoscopists. However, concordance of bowel preparation adequacy with a second observer seldom occurred. The full Ottawa preparation scale requires a total score out of 14 as a summation of three segments of colon and fluid quantity. Endoscopists in this study instead simplified the overall quality of bowel preparation from 0 to 4. This method, while simpler, may also not benefit from the nuance of the full Ottawa Preparation Scale in assessing bowel preparation quality. Furthermore, the Ottawa scale does not have a validated cut‐off value for what defines adequate bowel preparation and relies on clinical decision‐making to that effect. This study chose to define adequate bowel preparation as Excellent (0), Good (1), and Fair (2) classifications, but this is not a universally accepted definition and may vary between clinicians.

Repeat colonoscopies due to poor bowel preparation and prolonged procedure time carry risks for the patient. Anesthetic or procedural adverse events during or after the colonoscopy have been extensively investigated in literature.^36^, ^37^ Patients aged 65 years or older, the primary demographic undergoing colonoscopy, have been shown to be at an even higher risk of complications.^38^ Furthermore, as the incidence of colorectal cancer increases not only in Australia but worldwide,^39^ so does the health‐care burden and financial challenges of the disease.^40^ Poor bowel preparation has a substantial financial impact on the public system.^5^ A review by Bechtold *et al*. discusses different options with the aim to help endoscopists optimize visualization prior to colonoscopy.^41^ Identifying patients at risk of poor bowel preparation or prolonged cecal intubation allows for opportunities to minimize the harms of both. Interventions for these vulnerable groups, such as reinforcing adherence; educational videos; and, particularly, optimizing runway time, may serve to reduce the rate of missed lesions and repeat colonoscopies.
